# A randomized, double-blind, placebo-controlled trial to assess the efficacy of topiramate in the treatment of post-traumatic stress disorder

**DOI:** 10.1186/1471-244X-9-28

**Published:** 2009-05-29

**Authors:** Marcelo Feijó Mello, Mary Sau Ling Yeh, Jair Barbosa Neto, Luciana Lorens Braga, Jose Paulo Fiks, Daniela Deise Mendes, Tais S Moriyama, Nina Leão Marques Valente, Mariana Caddrobi Pupo Costa, Patricia Mattos, Rodrigo Affonseca Bressan, Sergio Baxter Andreoli, Jair Jesus Mari

**Affiliations:** 1 Department of Psychiatry, Universidade federal de São Paulo, São Paulo, Brazil

## Abstract

**Background:**

Topiramate might be effective in the treatment of posttraumatic stress disorder (PTSD) because of its antikindling effect and its action in both inhibitory and excitatory neurotransmitters. Open-label studies and few controlled trials have suggested that this anticonvulsant may have therapeutic potential in PTSD. This 12-week randomized, double-blind, placebo-controlled clinical trial will compare the efficacy of topiramate with placebo and study the tolerability of topiramate in the treatment of PTSD.

**Methods and design:**

Seventy-two adult outpatients with DSM-IV-diagnosed PTSD will be recruited from the violence program of Federal University of São Paulo Hospital (UNIFESP). After informed consent, screening, and a one week period of wash out, subjects will be randomized to either placebo or topiramate for 12 weeks. The primary efficacy endpoint will be the change in the Clinician-administered PTSD scale (CAPS) total score from baseline to the final visit at 12 weeks.

**Discussion:**

The development of treatments for PTSD is challenging due to the complexity of the symptoms and psychiatric comorbidities. The selective serotonin reuptake inhibitors (SSRIs) are the mainstream treatment for PTSD, but many patients do not have a satisfactory response to antidepressants. Although there are limited clinical studies available to assess the efficacy of topiramate for PTSD, the findings of prior trials suggest this anticonvulsant may be promising in the management of these patients.

**Trial Registration:**

NCT 00725920

## Background

Posttraumatic stress disorder (PTSD) is the only psychiatric disorder that has an etiologic component: exposure to a traumatic event. The disorder is characterized by three symptom clusters: reexperience of the event, avoidance/numbing, and hyperarousal resulting from exposure to a traumatic event[1]. Data from the National Comorbidity Survey in United States estimated a lifetime PTSD prevalence rate at 7.8% (10.4% for women and 5% for men) and to be more prevalent among women and the previously married. The trauma most likely to be associated with development of PTSD among men (69%) and women (45.9%) alike was rape. Survival analyses demonstrated that PTSD failed to remit in more than one third of persons even after several years of the occurrence of the traumatic event, demonstrating that PTSD tends to be a chronic disorder [2].

Furthermore, PTSD has a high comorbidity with other psychiatric conditions such as substance abuse/dependence, anxiety disorders, and major depressive disorder causing a worsening prognosis [2].

The development of treatments for PTSD is challenging due to the complexity of the symptoms and psychiatric comorbidities. Psychotherapy and pharmacotherapy are the two main classes of PTSD treatment. In the Cochrane systematic review, Bisson and Andrew [3] evaluated the efficacy of five categories of psychotherapeutic interventions: trauma-focused cognitive- behavioral therapy/exposure therapy (TFCBT), stress management, other therapies (supportive therapy, nondirective counseling, psychodynamic therapy, and hypnotherapy), group cognitive behavioral therapy and eye movement desensitization and reprocessing (EMDR). The authors analysed thirty-three studies and concluded that individual TFCBT, stress management, group TFCBT, and EMDR were more effective than wait list and other therapies. Bradley et al [4] conducted a multidimensional meta-analysis of psychotherapy studies published between 1980 and 2003. The meta-analysis included twenty six studies and reviewed exposure therapy, cognitive behavioral therapy (CBT), exposure plus CBT, EMDR and others. The authors found that exposure therapies, other cognitive behavior therapy approaches, and EMDR are efficacious in reducing PTSD, but found no significant differences between the various CBT modalities and between CBT and EMDR. Several review of psychotherapy have been published demonstrating that cognitive-behavioural and similar psychotherapies are effective in the treatment of PTSD.

The National Institute for Clinical Excellence (NICE) [5] recommended trauma focused psychological therapy as a routine first-line treatment for adults in preference to pharmacotherapy. In cases that drug treatment is required, paroxetine and mirtazapine were approved for general use, and amitriptyline and phenelzine for use only by mental health specialists. Although controlled trials with paroxetine did not show significant benefits on the main outcome variables, this is the only drug approved for PTSD in UK.

In a recently published systematic review, Stein et al [6] evaluated thirty five short term randomized controlled medication trials for PTSD (4597 participants). In thirteen trials, response to medication occurred in 59.1% of patients (644 participants), while response to placebo was seen in 38.5% of patients (628 participants). Significant reductions in symptom severity were observed for patients who received medications in seventeen trials. The mean total CAPS score for the medication group was 5.76 points lower than that for the placebo group (95% CI – 8.16 to -3.36 and 2507 participants). Evidence of treatment efficacy was most convincing for the SSRIs. Furthermore, medication was superior to placebo in reducing the severity of the three symptom clusters of PTSD, as well as alleviating the symptoms of depression, and in improving the quality of life measures. The current evidence base of randomized clinical trials is unable to demonstrate superior efficacy or acceptability for any particular medication class.

Nevertheless, the bulk of evidence for the efficacy of medication has been with the SSRIs and supports expert consensus guidelines that these medications constitute the first-line medication choice in PTSD.

The American Psychiatric Association [7] practice guidelines rated the SSRIs as first-line pharmacotherapy, with sertraline and paroxetine being US Food and Drug Administration approved for PTSD. The authors did not find efficacious pharmacological intervention to recommend in preventing the development of acute stress disorder or PTSD. The tricyclics antidepressants and monoamine oxidase inhibitors may also be beneficial (recommended with moderate clinical confidence) and the benzodiazepines, anticonvulsants, antipsychotics and adrenergic inhibitors may be recommended on the basis of individual circumstances. Trials of atypical antipsychotics olanzapine and risperidone found some evidence in terms of efficacy for PTSD with psychotic symptoms. Olanzapine, a second-generation antipsychotic agent, when prescribed to augment ongoing sertraline treatment, was shown to produce improvement in PTSD, depressive, and sleep-related symptoms in Vietnam veterans [8]. Open-label studies of adjunctive olanzapine have demonstrated symptom reduction in veterans with PTSD [9]. A small controlled study of risperidone in chronic combat related PTSD and comorbid psychotic symptoms demonstrated that reexperiencing and global psychotic symptoms were reduced [10]. Although the SSRIs are the mainstream treatment for PTSD, many patients do not have a satisfactory response to these antidepressants. The development of pharmacological treatments for PTSD is challenging due to the heterogeneity of symptoms. Recently, anticonvulsants have been evaluated for their potential efficacy for the treatment of PTSD. Open and randomized trials of carbamazepine [11], valproic acid [12,13], and lamotrigine [14] suggested that these agents may be efficacious in PTSD treatment.

The hypotheses on the etiology of PTSD have suggested that after exposure to traumatic events; the limbic nuclei may become kindled or sensitized, resulting in increased susceptibility to physiologic arousal [15,16]. The implication of the limbic kindling-like phenomenon in the pathophysiology of PTSD has stimulated clinical research in antiepileptic drugs in the treatment of PTSD.

Topiramate is an anticonvulsant with inhibitory activity in animal kindling models and with multiple mechanisms of action: inhibition of carbonic anhydrase, blockade of Na+ channels, inhibition of some high-voltage-activated Ca2+, negative modulating effect on the kainate/AMPA (α-amino-3-hydroxy-5-methylisoxazole-4-proprionic acid) subtype of glutamate receptors, ability to modulate NMDA (Nmethyl-D-aspartate) glutamate receptor and enhancement of GABAergic activity at GABA2 receptors [17,18].

The blockage or the stabilization of the AMPA (α-amino-3-hydroxy-5 methyl-4 isoxazole propionic acid) receptor may be a possible mechanism responsible for the increase in the threshold for flashbacks and nightmares that may explain the treatment response to topiramate in PTSD [19]. Several animal model studies show that AMPA antagonists attenuate acoustic startle response when administered into the amygdala. For instance, Khan and Liberzon [20] demonstrated that topiramate reduced significantly the acoustic startle response in rats after a single prolonged stress episode.

Glutamatergic neurons play a role in the neuronal plasticity associated with longterm potentiating in adaptation to stress [21]. Glutamate is the primary excitatory neurotransmitter in the central nervous system. Glutamate NMDA (N-methyl D-aspartate) receptors have been implicated in reconsolidation, and extinction of memories. The reconsolidation of spatial and contextual fear memory and odor reward association is impaired by NMDA antagonists [22,23]. Lee et al [24] demonstrated that the NMDA receptor antagonist (+)-5-methyl-10,11-dihydro-SHdibenzo [a, d] cyclohepten-5,10-imine maleate (MK-801) blocked and the agonist D-cycloserine (DCS) potentiated extinction of fear memory when they were administered before a long extinction training session whereas MK-801 impaired and DCS increased reconsolidation of memory when they were administered before brief memory reactivation session.

Although much of the research associate the glutamatergic systems with learning and memory; the GABAergic (γ-aminobutyric acid) system in the basolateral amygdala, is also involved in the acquisition and extinction of fear memories [25]. Reduced GABA function is involved in the formation of fear memories and the development of anxiety disorder. Stork et al [26] found that conditioned fear in mice is associated with a reduction of extracellular GABA levels in the amygdala indicative of a reduced GABA release and/or increased GABA uptake from the extracellular space.

Clinical studies with topiramate suggest that it is effective as monotherapy or adjunctive therapy in PTSD. Two open-label studies demonstrated topiramate to be effective and to have a rapid onset of action. Berlant and van Kammen [27] have reported that topiramate decreased nightmares in 79% and flashbacks in 86% of civilian patients with chronic PTSD with improvement of nightmares in 50% and of intrusions in 54% of patients with these symptoms. A partial response was reported for 95% of patients at a dosage of 75 mg/day or less and in 91% of full responders at a dosage of 100 mg/day or less. The nonhallucinatory subgroup achieved a higher response rate (89%). In a prospective open-label study, Berlant [28] treated thirty three civilians with chronic PTSD, with topiramate as monotherapy or augmentation therapy. The PTSD Checklist-Civilian Version (PCL-C) total symptoms declined by 49% at 4th week, with similar subscale reductions for reexperiencing, avoidance/numbing, and hyperarousal symptoms. Topiramate suppressed nightmares in 79% and decreased symptoms of intrusions in 94% in those patients with these symptoms. There is only one double-blind, placebo- controlled study of topiramate as a monotherapy in PTSD. Tucker [29] conducted a 12-week, double-blind, placebo-controlled evaluation of topiramate as a monotherapy in thirty eight civilian PTSD patients. Patients in the topiramate group exhibited significant reductions in reexperiencing symptoms (CAPS cluster B: topiramate = 74.9%; placebo = 50.2%; p = .038) and reductions approaching statistical significance, based on a nominal p value, were noted in mean total Clinical Global Impressions-Improvement Scale scores (topiramate = 1.9 ± 1.2; placebo = 2.6 ± 1.1; p = .055). Although there was a reduction in total Clinician-Administered PTSD Scale (CAPS) score from baseline (topiramate = -52.7; placebo = -42.0), the difference was not statistically significant (p = .232). But not all studies found positive results, Lindley [30] in a double-blind, placebo-controlled study using topiramate as an augmentation therapy in forty male veterans with PTSD did not find significant treatment effects versus placebo. However, there was a high dropout rate from the study with 11 (55%) topiramate and 5 (25%) placebo subjects not completing the seven week treatment, with 40% of topiramate and 10% of placebo dropping because of adverse effects. Patients in the topiramate group that completed the study, had a significant improvement in the reexperiencing subscale of the CAPS suggesting efficacy for the patients that continued the medication.

Extensive efforts have been made to establish guidelines based in evidence for PTSD treatment, however there is still no specific drug proved efficacious. Although there are limited clinical studies with topiramate in the treatment of PTSD, the findings of prior topiramate studies suggest that this anticonvulsant may be promising in the management of patients with PTSD.

### Aims of the Study

1) To compare the efficacy of topiramate with placebo in the treatment of PTSD. Clinical response will be considered when there are a 20% reduction on CAPS scale scores from baseline and remission when CAPS score is less than 19.

2) To study the tolerability of topiramate in the treatment of PTSD, through the dropout rate in each group.

### Research Goals

The trial will compare the efficacy of topiramate with placebo in the treatment of PTSD, and will study its tolerability through the drop out rate in each group. The primary hypothesis is that topiramate will be superior to placebo in the treatment of PTSD. Secondary research goals includes studying the efficacy of topiramate in social adjustment, functioning and quality of life. Additionally, evaluating treatment adhesion, and studying the efficacy of topiramate in depressive symptoms.

## Materials and methods

### Subjects

All elegible patients admitted at the outpatient clinic from the violence program of Federal University of São Paulo Hospital (UNIFESP) who read and signed a consent inform approved by the UNIFESP Institutional Review Board (IRB) will enter the study. A pre-request to be admitted at this program is having passed through a stressor event which imposed a risk on subjects' physical and/or psychological integrity (DSM-IV criterion A for PTSD diagnostic). Patients looked for an appointment at the program spontaneously, or through health worker recommendation or were called by phone or by mail from a list of subjects which had a PTSD diagnostic after a CIDI submission by trained raters on the Sao Paulo epidemiological catchment area study. This was an epidemiological survey of a random sample of 3000 participants living in the city of Sao Paulo to assess the relationship between exposure to violence and the prevalence of PTSD and common mental disorders.

All patients will be administered the Structured Clinical Interviews for DSM-IV Axis I and Axis II (SCID-I and SCID-II, respectively) by a trained psychiatrist [31,32]. Patients will be eligible if they meet the inclusion criteria: 1) DSM-IV criteria for a diagnosis of PTSD [1], 2) both gender, aged between 18 to 60 years of age. 3) Women of childbearing potential have to be practicing reliable contraception and cannot be pregnant or breast-feeding during the course of the study. The exclusion criteria will be: 1) lifetime history of bipolar, psychotic, borderline personality disorder; substance dependence or abuse (excluding nicotine and caffeine) in the previous 6 months; 2) serious or unstable concurrent illness; history of nephrolithiasis, 3) use of psychotropic medications for the previous 2 weeks (6 weeks for fluoxetine); 4) body mass index below 20; 5) current suicidal ideation or psychotic symptoms will be excluded from the study.

### Measures

• *The Structured Clinical Interview for DSM-IV-Axis I and II *(SCID-I and II) [31,32]: is a semi-structured interview that allows for the diagnosis of Axis I and II disorders, respectively, according to DSM-IV criteria [1].

• *The Clinician-Administered PTSD Scale *(CAPS) [33]: is a structured interview developed to diagnose PTSD and rate its severity. It is comprised of 30-items to assess PTSD-related symptom frequency and severity. Scores range from 0 to 136, with scores classified as follows: subclinical, from 0 to 19; mild, from 20 to 39; moderate, from 40 to 59; severe, from 60 to 79; extreme, 80 and above.

• *The Beck Depression Inventory *(BDI) [34]: is a 21-item self-report inventory designed to measure the severity of depression. Scores range from 0 to 63, with depression classified as minimal when scores range from 0 to 11, mild from 12 to 19, moderate from 20 to 35, and severe from 36 to 63.

• *MOS 36-Item Short-Form Health Survey *(SF-36) [35]: is a scale to measure mental and physical health concepts. It is a 36-item self-report instrument. The SF- 36 measures social functioning correlated to clinical conditions. As higher is the score, better is the quality of life. The instrument gave a global score, as specific areas scores: functional capacity, physical aspects, pain, general health condition, vitality, social aspects and emotional aspects, and mental health.

• *Social Adjustment Scale *(SAS) [37]: is a 54-item scale, ranging from 1 to 5 from normal to severely misadjusted. Used to evaluate social adjustment, normal score is around 1.56 (+/- .36).

• *Global Assessment of Functioning (GAF) Scale *[38]: is a single-item rating scale for evaluation the overall patient functioning during a specified period on a continuum from psychological or psychiatric sickness to health. The scale value ranges from 1 (hypothetically sickest person) to 100 (hypothetically healthiest person), divided into 10 equal intervals. A modified version of the Global Assessment Scale (GAS) was included in DSM-III as the Global Assessment of Functioning (GAF) Scale.

• *The Clinical Global Impression – Severity scale (CGI-S) *[37]: is a 7-point scale that requires the clinician to rate the severity of the patient's illness at the time of assessment, relative to the clinician's past experience with patients who have the same diagnosis. Considering total clinical experience, a patient is assessed on severity of mental illness at the time of rating 1 = normal, not at all ill; 2, borderline mentally ill; 3, mildly ill; 4, moderately ill; 5, markedly ill; 6, severely ill; or 7, extremely ill.

• *The Clinical Global Impression – Improvement scale (CGI-I) *[38]: is a 7 point scale that requires the clinician to assess how much the patient's illness has improved or worsened relative to a baseline state at the beginning of the intervention, and rated as: 1, very much improved; 2, much improved; 3, minimally improved; 4, no change; 5, minimally worse; 6, much worse; or 7, very much worse.

### Study design

This is a 12-week randomized, double-blind, placebo-controlled clinical trial that will be conducted at the Federal University of São Paulo Hospital (UNIFESP). The study was approved by UNIFESP Institutional Review Board (IRB), and will follow the Helsinky Declaration, and the local ethical laws. Ethical Committee: 196/96

#### Screening Phase

After the SCID-I and II administration, eligible patients will undergo a brief physical examination with height, weight, heart rate and systemic blood pressure measured and a clinical evaluation including electrocardiogram (EKG) and laboratory blood test for complete blood count, fasting glycemia, sodium, potassium, urea, creatinine, T3, T4, and TSH. Women will have a pregnancy test done, to assure they are not pregnant. The psychiatrist who administered the SCID, will also evaluate patients clinical conditions and functionality through CGI-S and GAF. The Clinician-Administered Posttraumatic Stress Scale (CAPS)[33] will be administered to the patients by trained raters, patients also will completed the Beck depression inventory (BDI) [34], the medical outcomes self-report (SF-36)[35] and the social adjustment scale (SAS) [36].

#### Wash-out phase

Patients will receive placebo pills and will be re-evaluated by the same psychiatrist one week after. Those having a significant improvement of the symptoms (a CGI-I score less than 3) will be excluded (avoiding premature placebo effects).

#### Active treatment phase

Those patients without improvement during the placebo trial, and do not show any laboratory and clinical condition exclusion criteria will be randomly assigned to receive either placebo or topiramate, in a 1:1 ratio using computer- generated code. The active and placebo pills will be identical. All research and clinical staff will be blinded to the randomized assignments.

Study medication will start at 25 mg/day once daily, at night, and will be increased 25 mg weekly, as tolerated, until complete or nearly complete efficacy is achieved or until 200 mg/day is reached. No other psychotropic medications will be allowed during the study, except for zolpidem (10 mg/day), if needed, for insomnia.

Study follow-up visits will be conducted at baseline, and weeks 1, 2, 3, 4, 6, 8, and 12.

Symptoms and side effects will be assessed, as well as measurement of systemic blood pressure and pulse rate in each visit by the investigator, who is blind to medication assignment. Compliance will be assessed by counting the amount of pills left in the bottle of medicine in each visit.

Treatment will be continued for 12 weeks (active treatment phase); then drug administration will be withdrawn gradually during 2 weeks with reductions of 25/50 mg every three days, according to the final dosage reached.

Patients will be assessed until completing 6 months during a follow-up period. If a patient showed a symptomatology worsening during the active treatment phase (12 weeks) when receiving active drug treatment, characterized by a one-point decrease at CGI -I or present serious adverse effects related to medication, he/she will be excluded from the study.

### Sample Size

The number of subjects calculated for the trial is 72, being 36 on each cell. The sample size was calculated considering a 5% probability of type I error (significance level), and 20% for type II error (power test of 80%), on a bi-caudal test. To the calculation we use an average of 60% for response from PTSD for active drugs, based on published literature, and 30% of placebo response.

### Statistical Analysis

All data will be analyzed by SPSS (version 13.0). The frequencies of nominal variables from all patients will be calculated. The data analysis will be conducted using a ANOVA comparing each scale during the 2 continuous measures from baseline and 12 weeks with a Between Subject factor was included as independent variable (treatment group). The confidence level that will be used for all comparisons will be 5% (p < .05). All observed Power as a type II error function will be presented with minimum values of significance for each comparison. CAPS scores from baseline and endpoint will be stratified according severity, as nominal data they will be compared using the chi-square test and the Fisher test when there were cells that counted less than five or at least one cell counted zero subjects. For missing data we will use the last observation carried forward strategy for an intention-to-treat analysis.

Efficacy analyses will be performed in the intent-to-treat population of patients who received at least one dose of topiramate. The primary efficacy endpoint will be the change in PTSD Scale- CAPS total score from baseline to the final visit at 12 weeks, which will be compared between treatment groups. Missing efficacy data at 12 weeks will be imputed with the last observation carried forward. The secondary outcomes between groups will be comparing the Clinical Global Impressions (CGI), the Beck Depression Inventory (BDI), the Global Assessment of Function (GAF), and the PANAS scales. Other secondary analyses will include an evaluation after 24-weeks (12-week after last patient visit with drug) data with endpoint analyses between the treatment groups covarying for baseline scores. On all secondary outcomes, the Bonferroni correction will be applied for testing multiple variables. Response will be defined as a 20% improvement in the baseline CAPS total or positive symptom scores at 12 weeks and will be compared between treatment groups using a chi-square test. Proportions of patients completing the 12-week double-blind study will be compared between treatment groups using a chi-square test. Adverse events reported by 10% of patients in each treatment group will be compared using a chi-square test. Laboratory values, vital signs and physical examinations are going to be compared from baseline and in reference to normal ranges, and reported as normal or abnormal. Changes in body weight (lb), body mass index (BMI; kg/m2) at last visit in the 12-week dataset will be compared as mean changes between treatment groups using t-tests.

## Competing interests

The authors declare that they have no competing interests.

## Authors' contributions

JJM, MFM, developed the design of the randomised clinical trial and participated in writing the article. MSLY is the principal investigator and writer of this manuscript. DDN, MSLY, JPF, NLMV, JBN, NMV and PM will participate as clinical psychiatrists. MCPC will be the study coordinator. RAB and SBA participated on the study design. All authors have read and approved the final manuscript.

## Pre-publication history

The pre-publication history for this paper can be accessed here:


